# Behavioural Profiles of Brown and Sloth Bears in Captivity

**DOI:** 10.3390/ani7050039

**Published:** 2017-05-13

**Authors:** Giovanni Quintavalle Pastorino, Yiannis Christodoulides, Giulio Curone, Paul Pearce-Kelly, Massimo Faustini, Mariangela Albertini, Richard Preziosi, Silvia Michela Mazzola

**Affiliations:** 1 Department of Veterinary Medicine, Università degli Studi di Milano, Via Celoria 10, Milan 20133, Italy; giovanni.quintavalle@unimi.it (G.Q.P.); giulio.curone@unimi.it (G.C.); massimo.faustini@unimi.it (M.F.); mariangela.albertini@unimi.it (M.A.); 2 The Royal Veterinary College, Royal College Street, London NW1 0TU, UK; yiannis40@gmail.com; 3 Institute of Zoology, Zoological Society of London, Regents Park, London NW1 4RY, UK; ppk@zsl.org; 4 Division of Biology and Conservation Ecology, School of Science and the Environment, Faculty of Science and Engineering, Manchester Metropolitan University, Manchester M1 5GD, UK; R.Preziosi@mmu.ac.uk

**Keywords:** *Melursus ursinus*, *Ursus arctos*, personality, behaviour, bear

## Abstract

**Simple Summary:**

Animal personality research is a growing field, since understanding animal personalities has notable implications in ecology and the evolution of animal behaviours. In the current study, we tested different methods described in the literature to obtain robust individual behavioural profiles. Data collected through behavioral observations were categorised into activity budgets, space usage, and social interactions for each individual. In addition, behavioural profile questionnaires were completed by the three zoo keepers who had regular interactions with the bears. The questionnaires included 22 adjectives, which were rated on a scale of 1–12 depending on how well they described each individual bear. The mean ratings of the keepers were used to create the behavioural profiles by adding the adjectives to the appropriate domains, according to the NEO Five Factor Inventory of personality model (NEO-FFI). The data gathered was used to produce behavioural profiles for all animals, in order to clarify the personality characteristics of each subject. Testing and improving existing methodologies to determine animal personality is important for providing optimal welfare and management of captive animals, since it can help to develop more effective management regimes in zoos by remodelling husbandry according to each animal’s personality type.

**Abstract:**

Three brown bear (*Ursus arctos arctos*) individuals and two sloth bear (*Melursus ursinus inornatus*) individuals were observed in captivity to produce behavioural profiles for each individual. Data collected through behavioural observations were used to produce activity budgets, and to identify space usage and certain aspects of social behavior. Behaviour monitoring allowed the researchers to evaluate the welfare of the animals by identifying the occurrence of stereotypic behaviours, which are sometimes associated with stress. Behavioural profiles were created using data obtained through behavioural observations (coding) and keeper questionnaires (rating). The behavioural observations indicated a number of stereotypic behaviours in sloth bears but not in brown bears. The uniformity of zone usage was calculated to investigate if the enclosure size and features were adequate for use, and a social aspect of otherwise solitary animals was also identified. The behavioural profiles generated through coding and rating were compared to determine the reliability between these two methods in Ursids. Profiles were not compared between individuals since this study is not a comparison between different personality types but rather an effort (one of the few ones existing in literature) to select a valid and reproducible methodology capable of assessing personality in bears.

## 1. Introduction

Animal personality has been studied for some time now, but recently it has increased in interest as more researchers accept the presence of personality in animals [1,2,3]. Moreover, understanding animal personalities has notable implications in the ecology and evolution of animal behaviours [4,5,6]. As noted by Chadwick [7], there is inconsistency in the literature regarding the terms used when describing animal personality [8,9,10,11]. Many researchers refer to “temperament” [12], others to “behavioural profiling” [13], and still others refer to “individual differences” [14,15] or “individual distinctiveness” [16]. In the current study, “temperament” and “personality” have the same meaning as proposed by Réale et al. [8] where personality is the variation in behaviour between individuals in a population that is consistent across time and context. One method to evaluate personality is to acquire trait ratings from the people who know the animals best (keepers, owners) to assess the personality of the animals they attend [17]. Another approach is by behavioural coding, where objective data is recorded during behavioral observations, either under normal circumstances or in response to specific tests [7]. In the current study, adaptations of these methods will be applied in order to delineate behavioural profiles. Thurstone [18], in a famous study of human psychology, was the first to divide a number of traits into five groups that were representative of personality, and those specific traits were grouped as such to exemplify each group. Costa and McCrae [19] further developed the concept by creating the NEO Five Factor Inventory of personality model (NEO-FFI), which allowed traits to be grouped so as to form five dimensions of human personality. These domains/dimensions were: openness or closeness to experience (O+/O−), conscientiousness or lack of direction (C+/C−), extraversion or introversion (E+/E−), agreeableness or antagonism (A+/A−), and neuroticism or emotional stability (N+/N−). This was originally created and used for humans but as research started focusing on animal personality, these traits were also used to infer animal personality as well [20,21]. The NEO-FFI was adapted for use in non-human animals using traits more suitable to animals [22]. After the initial adaptation, other studies have tried to adapt the NEO-FFI to other species [23] by developing specific questionnaires, such as the Hominid Personality Questionnaire for animals [24]. In the current study, the personality domains adapted for animals will be used in an effort to create behavioural profiles for each individual [22,25]. Personality studies are important because they can help develop more effective management regimes in zoos and increase individual animals’ welfare by remodelling husbandry according to their personality type [26,27]. There is evidence that personality has a prominent role in determining the reproductive success of a number of species in zoos. A study by Chadwick [7] examined the effect of personality on cheetah reproductive pairs, promoting the use of personality profiles as a tool to match individuals in order to have higher reproductive success [27]. The ability to forecast a potential successful match before the relocation of animals occurs increases the welfare of the animals, since it minimizes the chance of potential mismatches, which results in low or no reproductive success [7,28]. Moreover, in order to minimize the impact on the existing population dynamics, prior to introducing a new individual into an existing animal group, a study on personality profiles could be extremely useful, since it could help optimise the introduction strategy [27]. Similar benefits could also be realised in reintroduction programmes. Besides genetic heterozygosity and inbreeding avoidance, which are fundamental in the selection of individuals, a pre-release personality profile could help to infer the fitness of the individual in the proposed location [29]. This is particularly important for individuals with specific personality traits that cause them to respond inappropriately in stressful or dangerous situations [30]. Furthermore, there is evidence that in order to enhance the chance of survival, the founder population should contain a mix of personality profiles when reintroducing a species [31].

Sloth bears are classified as vulnerable in the International Union for Conservation of Nature (IUCN) Red List of threatened species and there are no reliable wild population estimates or population trend estimates for this species. The Brown bear is the most widely distributed ursid, and the total number of individuals is estimated to exceed 200,000. In the IUCN Red List of Threatened Species, Brown bears are classified as least concern [32].

According to the Zoological Information Management System (ZIMS) (the online database of wild animals maintained in captivity) there is a total of 713 *Ursus arctos* bears housed in 197 zoological institution and 263 *Melursus ursinus* bears, housed in 49 institutions. 

This is a pilot study on captive animals, where different methods used in the literature were tested for validity and reliability, in regards to brown and sloth bears [23]. Currently, only one research paper on wild bear personality has been published [33]. The authors found that individual brown bears behaved differently from each other. They suggested that consistent behavioural differences implied that each bear had its own distinct personality. Our study is the first one describing ex situ Ursids behavioural profiles. Captive animal studies provide information on the specific animals in the zoo in terms of their welfare, health, and management, as well as the exhibition of natural behaviours [34]. Ideally, a captive population will exhibit the behaviour it would have in the wild [35]. In addition to the natural behaviour repertoire, captive animals might exhibit some unnatural behaviours, i.e., defined stereotypic behaviours. Among others, these include: pacing, head rolling, over grooming, and self-directed behaviours, all of which are considered to be caused by the stress of the artificial settings of the environment [34]. Bear species have been found to be exceptionally prone to stereotypical behaviours [36,37,38], with sloth bears in particular being very susceptible to pacing [37]. Simple observational studies can be used to create activity time budgets to ascertain if wild and captive behaviours are similar and to observe any possible stereotypical behaviour that may affect the welfare of the animal [39]. Furthermore, a behavioural study can potentially indicate the enclosure usage by the animal in order to identify if it is evenly used and properly structured [40]. Following Rose and Robert [41], Spread of Participation Index (SPI) results may suggest the necessity to enhance the unused area with biologically relevant structures that induce animals to use these areas. In social animals, social interactions may be considered as environmental enrichment, since they could improve the welfare of the animals [42]. In the case of solitary species, housing more than one individual could also be beneficial to the animals and, as Yoerg’s [43] experiments on kangaroo rats (*Dipodomys heermanni*) have shown, even though solitary species spent more time on their own, they still have social interactions with neighbouring individuals.

The primary focus of the current study was to establish behavioural profiles for each individual, using both questionnaires and behavioural observations, assessing the reliability and the validity of the two methods. Moreover, it aimed to assess the welfare, health, and management of the animals, through the analysis of activity budgets and zone usage, and outline social interactions. Although the current study specifically focuses on Eurasian brown bears and Sri Lankan sloth bears housed at Whipsnade Zoo, these methodologies can also be used as guidelines to evaluate, and improve, the welfare and management of ex situ housed Ursids in general. In addition, since there is extremely limited literature on bear personality, this study can be the starting point in developing an effective methodology for creating personality profiles in bears. This study is intended to inform and encourage further personality research, and to provide a reliable and practical tool for the quick assessment of zoo animals.

## 2. Materials and Methods

### 2.1. Study Area and Animals

All the animals enrolled were from Zoological Society of London (ZSL). Whipsnade Zoo in the United Kingdom. Two species of bears were used in this study: the Sri Lankan sloth bears, *Melursus ursinus inornatus,* and the Eurasian brown bears, *Ursus arctos arctos* (Table 1).

**Figure 1:**
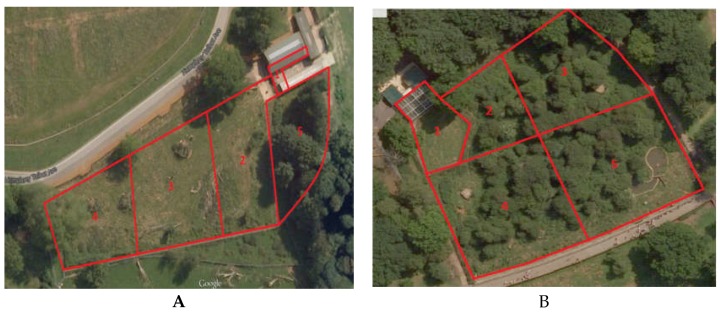
(**A**) Sloth bear enclosure and (**B**) brown bear enclosure. The pictures show the enclosures’ division into zones, used for zone usage and the Spread of Participation Index (SPI).

The sloth bears were kept in an enclosure (Figure 1A) built as an outside paddock with access to an inside enclosure. Part of the outside enclosure was a dense forest area and the remainder was an open, grassy area with small hills and a large wooden structure that the bears could climb on. Food was scattered or placed in enrichment objects about every two hours on a fixed schedule, and enrichment items were also placed in the enclosure every two to three days. The overall area of the enclosure was about 5695 m^2^.

The brown bears’ enclosure (Figure 1B) consisted mostly of an outside paddock with a small inside enclosure. The outdoor area comprised a dense forest with shrub vegetation, and included a number of dens and a small shallow pool for drinking and bathing. The overall area of the enclosure was about 5503 m^2^.

### 2.2. Observational Data

Bears were monitored three days a week for seven weeks, with four 50 min sessions each day, two morning sessions (between 10:00–12:00) and two afternoon sessions (between 14:00–16:00). The sessions alternated between the two species so that all the animals had equal time in each session slot. Focal sampling was used for this observation, choosing the sampling order randomly and state behaviour was recorded at 1 minute intervals [44]. Instantaneous sampling of event behaviours were recorded during the minute as they happened. During each session, time was split evenly on each animal, allowing 25 min for each sloth bear and 17 min for each brown bear. This methodology ensures that each animal’s observation session is independent from the other animals’ sessions.

The enclosures were arbitrarily divided into sections (Figure 1) in order to observe the zone usage of each animal. Enclosure zone data, recorded on the same time schedule as the behavioural data, were used to calculate the Spread of Participation Index for each individual [40].

Instantaneous sampling of close proximity data, recorded every minute, pointed out when each individual was within 7 m of another individual during one of the observations. The total number of close proximity events was compared to the probability of a chance encounter [7].

Before starting the scheduled behavioural observation sessions, an ethogram was created, integrating the data published in the literature [33,39,45,46,47] with the behaviours observed during some preliminary sessions. Behaviours were grouped in categories (Table 2) [22]. Activity budgets for each individual were created using observational data.

### 2.3. Behavioural Profiles

According to the NEO-FFI domains adapted for animals by Highfill and Kuczaj [22], behavioural profiles were created using the two methods. Conscientiousness was removed, as it is difficult to apply to non-primate animals [17].

In the first method, called behavioural coding, the behaviour of the animal is used to create a profile based on the behavioural observations, which are grouped in appropriate domains (Table 3) using an adaptation of the procedure used by Birgersson [25]. Foraging and eating were used in two instances since it was considered to be both exploratory behaviour and a behaviour that shows activity.

The second method involved trait ratings, with information provided by the zoo keepers. Questionnaires were given to all the zoo keepers who have regular interactions with the bears, following the protocol from Chadwick [7]. In order to describe behavioural and personality aspects, the questionnaires included 22 adjectives, which were rated on a scale of 1–12 depending on how well they described each bear. Three keepers completed the questionnaires for each animal. The mean ratings of the keepers were used to create the personality profiles by adding the adjectives to the appropriate domains (Table 4).

### 2.4. Statistical Analysis and Presentation of Data

All of the statistical analysis and calculations were done using the programs Microsoft Office Excel 2007 (Microsoft Italia, Milano, Italy) and Minitab 16 (GMSL S.r.l, Nerviano, Milano, Italy).

The SPI was used to see if the enclosure was used evenly by the animals or if there was skewed zone usage as described by Plowman [40]:

$$SPI=\frac{\sum |fo-fe|}{2(N-{fe}_{min})}$$

where “*fo*” is the observed frequency of observations in a specific zone and “*fe*” is the expected frequency (calculated, following [40] with “*fe*_min_” being the expected frequency of the smallest area). *N* is the total number of observations.

The probability of a chance encounter was calculated as described by Chadwick [7]. In the present study, any distance below 7 m was considered to be a close proximity event. This was modified from the original methodologies used by Chadwick in his cheetah study [7], which considered a close proximity event at a distance of 5 m, in order to account for the bigger dimensions of the bears. The random chance value was then compared to the observational encounters using a Chi-squared test.

Statistical analysis was done separately for behavioural coding and behavioural rating, since they produced two personality profiles. The Kruskal-Wallis test was then used to estimate the significant difference between the domains of each animal and the post hoc Bonferroni test revealed which domains were significantly different from the others.

Inter-rater reliability of the trait ratings by the zoo keepers was tested using Kendall’s coefficient of concordance since there were more than two raters.

The behavioural groups used in the activity budgets were also statistically tested using the Kruskal-Wallis test with the Bonferroni post-hoc test to ascertain what activities the individuals performed more frequently.

### 2.5. Ehtical Statement

All keepers gave their informed consent for inclusion before they participated in the study. The study was conducted in accordance with the Declaration of Helsinki, and the protocol was approved by the Ethics Committee of the Zoological Society of London (ZPD WAB7).

## 3. Results

### 3.1. Activity Budget

**Figure 2:**
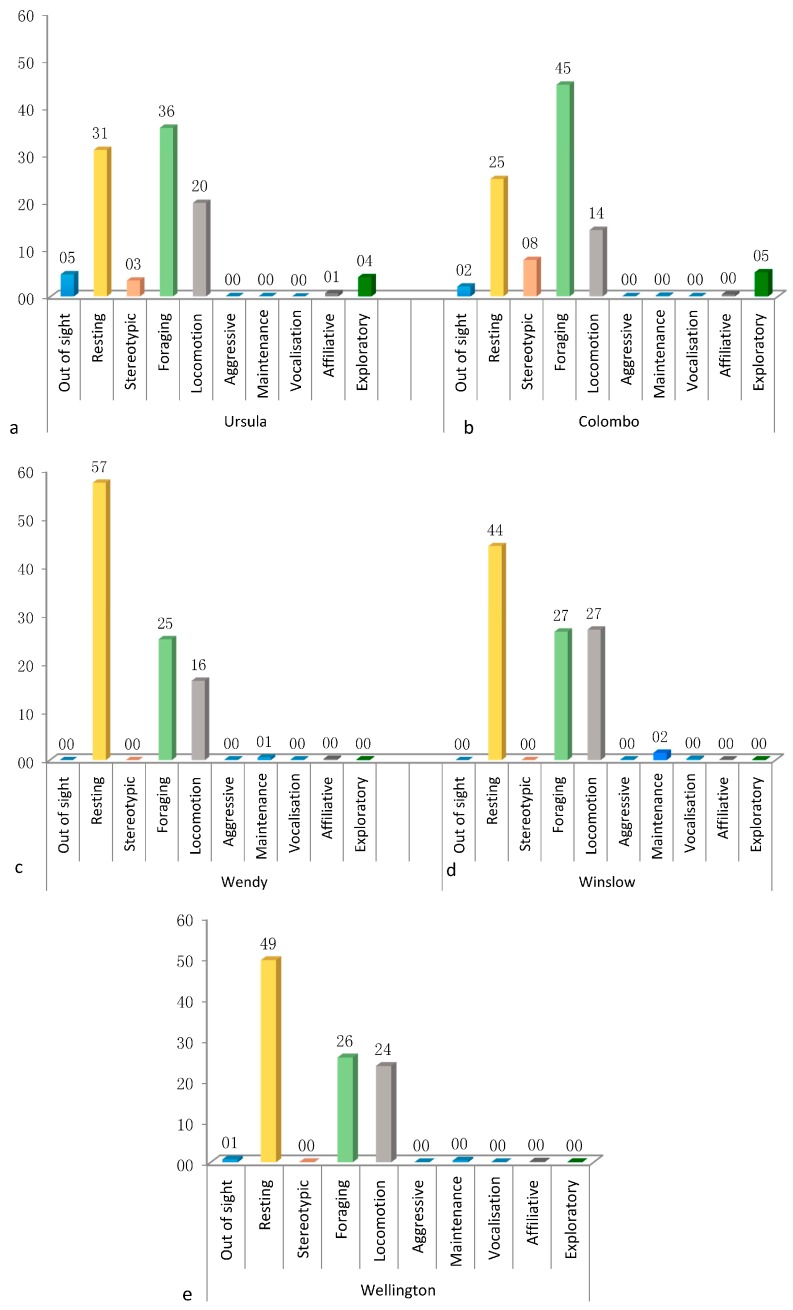
Column charts showing the activity budget of all individuals that were observed: (**a**,**b**) are sloth bears and (**c**–**e**) are brown bears.

Activity budgets for each animal were created using the observational data (Figure 2). In order to standardize the observation, the data was expressed in percentage form. Significant variation in the activity budgets exists in all the individuals’ behaviours, with the Kruskal Wallis test H ranging from 204.53 to 254.70 (*p* < 0.05).

Both bear species spent most of their time foraging and eating, moving around the enclosure, or being inactive. However, we can see from the table and the charts that brown bears spent almost half their time being inactive while the sloth bears spent approximately the same amount of time foraging and eating. In both bear species, behaviours like maintenance, aggression, affiliation, or being vocal were observed at a very low frequency or none at all. These were all statistically verified with the Bonferroni post hoc test (*p* < 0.05).

### 3.2. Zone Usage

The zone usage of each animal was noted during the observations and then used to calculate the SPI (Table 5).

The SPI has a range of 0 to 1 where 0 means the individual is using the enclosure evenly and 1 means that they use just one area of the enclosure; anything above 0 indicates a variable degree of unevenness. The difference between the expected and the observed usage, a by-product of the SPI calculation, is indicative of where there was higher or lower usage [40].

The mean SPI for the sloth bears is 0.365 while the mean SPI for the brown bears is 0.310, showing that the usage of the zones was unequal. The difference between the observed and expected values allows us to see which zones each individual used more and which they used less. The sloth bears used more zones 1 and 2 more often and zones 3, 4, and 5 less often. The brown bears used zones 1 and 4 more often and zone 2, 3 and 5 less often.

### 3.3. Probability of Chance Encounter

For sloth bears, the expected value for close proximity chance encounters in the sampling period was 100 times, while the effective observed frequency of encounters was 892. This difference was found to be statistically different (χ^2^ = 6586.27, *p* < 0.05), showing that sloth bears were found together more often than expected by chance. The brown bears’ expected frequency was 306 while the observed frequency of encounters was 551, which again was statistically different (χ^2^ = 228.85, *p* < 0.05), showing that they were found together more than expected. For the brown bears, Wellington and Winslow, we calculated with whom they spent more of their time in close proximity: Wendy, who was unrelated, or their sibling. Wellington spent more time with Winslow than Wendy (χ^2^ = 20.455, *p* < 0.05) and Winslow spent more time with Wellington than Wendy (χ^2^ = 19.360, *p* < 0.05).

### 3.4. Behavioural Profiles

Behavioural profiles are presented on one individual at a time. Each individual has a behavioural profile created using the observations and a profile created with the trait ratings. The domains, as mentioned in the introduction, will be used as O+, O−, E+, E−, A+, A−, N+, and N−. Where specific domains are mentioned to be significantly different than others, it was always with a *p* < 0.05.

#### 3.4.1. Ursula

**Figure 3:**
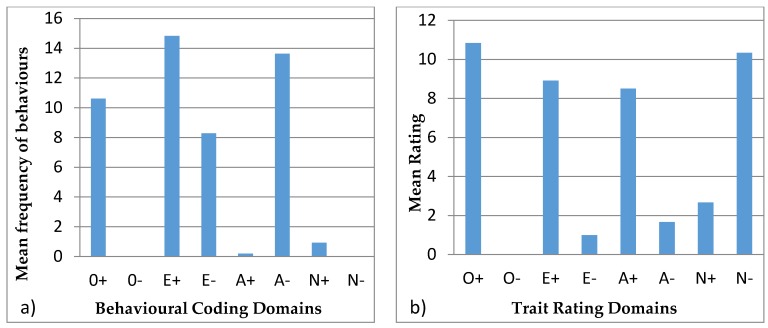
Behavioural profiles showing the domains of Ursula: (**a**) was created using observations (behavioural coding) and (**b**) was created from the questionnaires (trait ratings).

Figure 3 shows the behavioural profiles of Ursula. In the behavioural coding profile, the domains were found to be statistically different (H = 210.55, *p* < 0.05) and the post hoc Bonferroni test showed that the domains of O+, E+, E−, and A− are statistically different from the domains of O−, A+, N+, and N−. The trait rating profile again showed a significant difference (H = 22.14, *p* < 0.05) and the post hoc Bonferroni test showed that the significantly different groups were O+ with O− and E−, and there was a difference between N− and O−.

#### 3.4.2. Colombo

**Figure 4:**
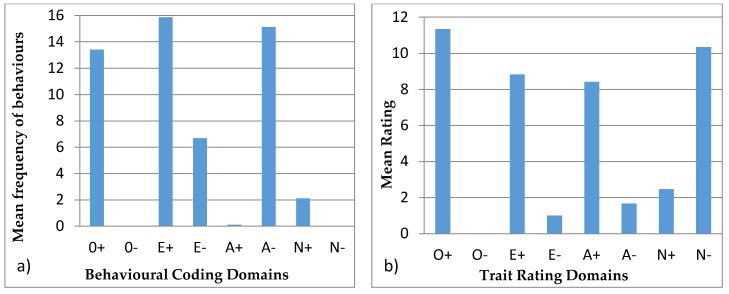
Behavioural profiles showing the domains of Colombo: (**a**) was created using observations (behavioural coding) and (**b**) was created from the questionnaires (trait ratings).

Figure 4 shows the behavioural profiles of Colombo. The behavioural coding profile showed statistical difference between the domains (H = 208.27, *p* < 0.05) with the post hoc Bonferroni test showing significant differences for O−, A+, N+ and N−, with each one being significantly different from the domains of O+, E+, E−, and A−. E− was also significantly different from A− and E+. The trait ratings profile showed a statistical difference (H = 22.06, *p* < 0.05) and the post hoc Bonferroni showed statistical differences in O+ with O− and E−, and difference between O− and N−.

#### 3.4.3. Wendy

**Figure 5:**
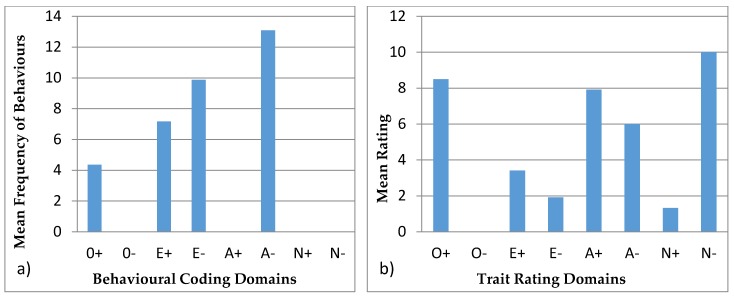
Behavioural profiles showing the domains of Wendy: (**a**) was created using observations (behavioural coding) and (**b**) was created from the questionnaires (trait ratings).

Figure 5 shows the behavioural profiles created for Wendy. The behavioural coding profile showed statistical difference between the domains (H = 222.79, *p* < 0.05) with A− being statistically different from all domains except E−, which is in turn statistically different from the rest, except for E+. E+ and O+ are statistically different from the remaining domains. For the trait rating profiles, there was statistical variation (H = 39.99, *p* < 0.05) with the group O− being different from N−, O+, A+, and A−. O+ and N− are also statistically different from E− and N+.

#### 3.4.4. Wellington

**Figure 6:**
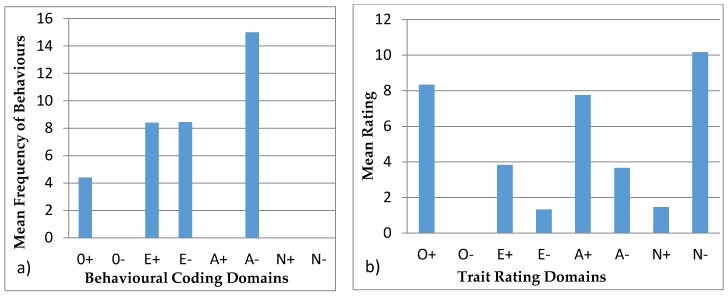
Behavioural profiles showing the domains of Wellington: (**a**) was created using observations (behavioural coding) and (**b**) was created from the questionnaires (trait ratings).

Figure 6 shows the behavioural profiles of Wellington. The behavioural coding profile domains were found to be statistically different (H = 261.30, *p* < 0.05) and the post hoc Bonferroni test showed that O+, A−, E+, and E− are statistically different from every other domain. Furthermore, A− and O+ are statistically different from E+ and E−. The trait rating profile again showed a significant difference (H = 60.82, *p* < 0.05) and the post hoc Bonferroni test showed that the significantly different groups were O+ and N− with O−, E−, and N+. Also, there was a significant difference between O− with A+, A−, and E+, and in A+ with N+ and E−.

#### 3.4.5. Winslow

**Figure 7:**
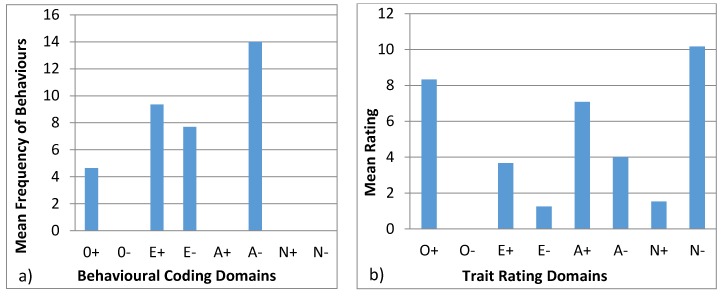
Behavioural profiles showing the domains of Winslow: (**a**) was created using observations (behavioural coding) and (**b**) was created from the questionnaires (trait ratings).

Figure 7 has the behavioural profiles of Winslow with the behavioural coding profile having statistical variation (H = 261.90, *p* < 0.05) with A−, E−, E+, and O+ being statistically different from O−, A+, N+, and N−. A− is also statistically different from O+ and E−, while E+ is different from O+.

Inter-rater reliability was statistically tested using Kendall’s coefficient of concordance (W) for every trait. For the sloth bears, the mean W was 0.704 with only two traits below 0.5. The standard deviation ranged from 0 to 3.06 for all the traits in all of the sloth bears. In the same respect, the brown bears had a mean W of 0.771, with only two traits below 0.5. The standard deviation ranged from 0 to 2 for all the traits in all of the brown bears.

## 4. Discussion

### 4.1. Activity Budget

The activity budgets (Figure 2) created using the observational data showed that both species of bears spent most of their time exhibiting behaviours that fall into the inactive, foraging and eating, or locomotion categories. Brown bears spent almost half of their time being inactive while the other half was split between locomotion, foraging, and eating. This is compatible with what other studies have shown on the activity budgets of brown bears, both in the wild and in captivity [33,37,45]. Behaviours like affiliation or aggression were rarely observed; this could be due to the fact that all the brown bears are female, thus having lower aggression displays [33]. As for affiliation, bears tend to be solitary animals, so low affiliation observations are expected [33,37,38]. A Montaudouin and Le Pape study [37] has shown that a large number of bears show stereotypic behaviour due to the small or unnatural enclosures or the absence of landmarks such as pools or ponds. In the current study, brown bears, which have spent their entire lives in a naturalistic enclosure, exhibit no stereotypic behaviours, such as pacing or swaying. This is considered to be a positive sign of their welfare, since stereotypic behaviours have also been found to be coping mechanisms that can essentially become habit and may eventually be exhibited in the absence of any stressor that may have been present when the behaviour was first exhibited [48].

Sloth bears spent more time foraging and eating than performing any other behaviour, with the rest of the time budget split between inactivity and locomotion. Furthermore, a small percentage of their activity was spent on other behaviours, like affiliation and exploration. This is consistent with behaviour observed in the wild and desirable ex situ situations [36,46].

The sloth bears exhibited stereotypic behaviour in the form of pacing, which accounted for 3–8% of their daily activity. Since, as described in the ecological and evolutionary biology literature, stereotypic behaviour can be also adaptive depending on environmental variation and predictability [4], in order to decrease stereotypic behaviours, scatter feeding or other environmental enrichment techniques are suggested [36]. In the ZSL Whipsnade zoo, environmental enrichment administered to sloth bears is abundant, in the form of scatter feeding occurring up to 10 times throughout the day, in order to keep them active and foraging. Moreover, they also receive a number of enrichment items that are constantly changed on a regular basis.

Brown bears in this study, showing no apparent stereotypic behaviour, are fed at a specific location once or twice a day and have the large, densely forested natural enclosure with a pool as their only form of enrichment. According to Montaudouin et al. [37], most bear species exhibit stereotypic behaviour in various forms, but sloth bears are notorious for their stereotypic behaviour based on observed stereotypes [36].

Almost all of the pacing observed was next to the keeper facilities, which could be the reason behind the stereotypic behaviour. Between bouts of pacing the sloth bears were “waiting” for the keepers to get the food into the enclosure. Such feeding related anticipatory behaviour has been observed in other species [49,50]. Although anticipatory behaviour is not considered stereotypic behaviour, it is possible that the two are related and anticipatory behaviour still decreases the welfare [50]. Another explanation for the observed anticipatory behaviour may be the diet of each animal: sloth bears rely almost completely on the food provided by their keepers, since they have not been observed eating grass or roots in their enclosure. On the other hand, brown bears eat roots and even graze at times so they can feed on the vegetation found in their enclosure, without having to wait for a keeper to bring them food, resulting in less stereotypic behaviour.

### 4.2. Zone Usage

The use of SPI (Table 5) has helped to calculate how evenly the bears used their enclosure [40]. The mean SPI for each species was moderate (0.301 and 0.365) showing that the bears did not use their enclosure as evenly as possible. Taking into account the difference of the expected and observed usage of each zone, it is evident that sloth bears were observed more in zones 1 and 2 whereas brown bears were observed more in zones 1 and 4. Rose and Robert [41] state that animals seem to prefer certain areas over others based on their biological importance. This may explain the zone usage in the current study. In both species, zone 1 is where the indoor enclosure is located and also where they receive part of their feeding. The brown bears are also fed in zone 4, which explains their preference for that zone as well. Regarding the sloth bears, zone 2 is where the keepers’ entrance is, so they have an inducement to spend time in that area in order to see when the keepers come with their food. Significantly uneven use of an enclosure suggests that the area or areas of the enclosure are insufficiently attractive to the animal, with the result that the suitable enclosure space is reduced. By improving the attractiveness of such enclosure areas, the animal can fully utilise the available enclosure space. Since the SPI value is below the average, and based on the aforementioned considerations, we do not suggest a change in the enclosure design in this instance [41].

### 4.3. Probability of Chance Encounter

Using the random generated points, we calculated that both species were found in close proximity significantly more than would be expected by random chance. The reason behind the expected random chance is that bears are solitary species and thus, even though in the current study the bears within the enclosure were either related to each other and/or had been living together most of their lives, they would spend more time on their own than in close proximity with other individuals. A study by Perret and Predine [51] measured cortisol levels in the solitary species of grey mouse lemurs, *Microcebus murinus*, and showed that cortisol levels were higher when the individuals were housed socially. However, a certain social aspect is present in all vertebrates, including solitary animals, given that each individual has adequate space for solidarity [34]. In this case, the sloth bears are siblings, which causes them to associate more than they would if they were not related [52]. Two of the brown bears are siblings, while the third one is not related to them. The bears have been living together all their life, since they were all born and raised together. This means that the unrelated brown bear is extremely familiar with the others, so this probably makes them feel comfortable being in close proximity [52]. However, the siblings spent significantly more time together than they did with Wendy, who is the unrelated bear. 

### 4.4. Behavioural Profiles

Behavioural profiles from behavioural coding described in the literature are usually paired with tests, such as mazes for exploration, mirror tests, or anti-predator behaviours. These tests require alterations to the environmental conditions of the animal, which are not always applicable or viable in zoos. These tests help identify specific personality characteristics, validating the profiles [21]. Hence, our study aims at creating a methodology, where behavioural observations can be used to infer personality traits. This approach minimizes animal disturbance and avoids invasive tests and also has the advantage that it can be easily reproduced. Réale et al. [53] argue that sampling normal behaviour to create personality profiles is subjective, since some behaviours are not easily classified into personality traits. However, conducting research on the matter can assist in identifying which behaviours apply to each personality trait, and thus create valid personality profiles. In certain animal groups like felids, research is more intensive [7,54] which allows for behaviours to be more robustly applied to personality traits. Moreover, felids have more expressive behaviours: they use different body stances and vocalisations, as well as facial, ear, or tail movements, and positions to show their behaviour and feelings, such as vigilance, anger, fear, submission, and affiliation, at the time [54,55]. On the contrary, bears are not so expressive, and females have less affiliative behaviour since they are solitary species [33,37,38]. None of the animals in this study exhibited very low frequencies of affiliative, aggressive, and vocal behaviour (Table 5). In addition, domains like N− and O− used for the behavioural profiles were not used in behavioural coding profiles (Figure 3, Figure 4, Figure 5, Figure 6 and Figure 7) since there were no suitable behaviours for use in those domains. Other domains like A+, A−, and E− obtained from the behavioural coding profiles seem to be very different from the ones obtained by the trait rating profiles. This could be due to the fact that some behaviours were used for one domain instead of another and thus, in order to be able to distinguish which behaviour belongs where, more research has to be done. Moreover, domains like O+, E+, and N+ seem to be more consistent since they have similar impact in both profiles. Using statistical tests like the Spearman’s rank correlation, we can see how the domains created using trait ratings can be correlated with the domains using behavioural coding, so as to merge the two at a later stage in order to achieve a more complete personality profile [56]. However, this requires a much larger sample size than we had in this study in order to get statistically significant results [7].

Furthermore, this current study on bears has a more reliable trait rating than the behavioural coding personality profile. That is, trait ratings have been used in a number of studies [7,22,33], which tested their reliability and validity. A study by Vazire et al. [57] argued that trait ratings are more reliable indicators of personality if the raters are knowledgeable about their animals. Highfill et al. [56] agreed that trait ratings are more reliable as long as the relationship between the animal and the raters is the same (i.e., all keepers), as was applied in this study. Inter-rater reliability was statistically evaluated and found to be reliable for the majority of the trait adjectives (20). The ratings also had a maximum standard deviation of 3.06 for sloth bears and 2 for brown bears, showing that the keepers agreed on the animals’ personality traits, which gave reliability to the results.

## 5. Conclusions

In the current study, the behavioural profiles were not created with the primary intention of comparing between individuals, but to provide a viable methodology that can be applied to a number of bear species. A valid methodology can help advance personality research and contribute to animal management and welfare assessments. In turn, the information gathered from personality research will help identify how an animal will react to new environments as well as to new individuals with specific personality profiles. This will aid bear conservation efforts, in terms of captive breeding and reintroduction success. Further study is needed to examine how different personality profiles interact with each other and to correlate the observed behaviours with the underlying personality traits, in order to predict individual behavioural responses to specific scenarios. In conclusion, this study incorporates behavioural sampling with personality profiling in a zoo environment and informs the wider animal personality research effort. 

## Figures and Tables

**Table 1:** Animals involved in the study.

Name	Species	Sex	Born	Born in	Relationship to Other Study Animals
Wendy	Brown Bear	Female	26/03/1991	Captivity	None
Wellington	Brown Bear	Female	15/01/1993	Captivity	Sister of Winslow
Winslow	Brown Bear	Female	15/01/1993	Captivity	Sister of Wellington
Ursula	Sloth Bear	Female	04/02/2001	Captivity	Sister of Colombo
Colombo	Sloth Bear	Male	05/01/1998	Captivity	Brother of Ursula

**Table 2:** Categories of behaviours exhibited by the bears during observations (adapted from Highfill [22]).

Behaviour	Description
Inactive	Bear is lying, sitting, standing on four paws or upright on two paws whether on land or water. Lying can be on the side, stomach, or back.
Foraging and Eating	Bear is actively searching and consuming food. This includes digging to get to food. Distinction between foraging and eating is not made because handling time is minimal or absent in some cases.
Locomotion	Movement of the bear like walking on land and water, running or climbing trees or other structures.
Stereotypic	Behaviour not exhibited in the wild. In this case it is pacing, which is walking repeatedly, for more than 30’, along the same path.
Aggression	Aggressive displays towards conspecifics or people.
Maintenance	Natural somatic behaviours like drinking water, urinating, defecating, grooming, or scratching.
Affiliation	Positive/friendly behaviours towards conspecifics like playing, sniffing, and rubbing.
Exploration	Interacting with the environment whether handling, sniffing, or rubbing against objects or parts of the enclosure.
Vocal	Sounds emitted by the bears to show danger, alarm, anger, or intimidation like barking and growling.

**Table 3:** Each domain has a positive and a negative part and behaviours were assigned to each one. Some had no corresponding observed behaviours.

Openness to Experience	Extroversion	Agreeableness	Neuroticism
+	+	+	+
Exploration	Vocal	Affiliation	Aggression
Foraging and eating	Locomotion		Stereotypic
	Foraging and eating		
−	−	−	−
	Inactive	Solitary	

**Table 4:** Adjectives and domains used for trait ratings, based on the human Five Factor Model. Except for *Openness to experience*, which was the only one with no corresponding negative adjectives, each domain has a positive and a negative part.

Openness to Experience	Extroversion	Agreeableness	Neuroticism
+	+	+	+
Curious	Active	Friendly to conspecifics	Aggression to conspecifics
Smart	Playful	Friendly to keepers	Aggression to familiars
	Vocal	Friendly to familiars	Aggression to unfamiliars
	Excitable	Friendly to unfamiliars	Tense
			Eccentric
−	−	−	−
	Fear of conspecifics	Solitary	Calm
	Fear of familiars		Self-Assured
	Fear of unfamiliars		
	Insecure		

**Table 5:** Table showing zone usage for each individual and each zone. *N* is the observed frequency of the individual in that zone and *D* is the difference between the expected and the observed frequency. A negative value indicates less usage than expected and a positive value indicates more usage than expected. SPI is the spread participation index with a scale of 0–1, with 0 being even usage and 1 being usage in one area.

Species	Animal	Zone 1		Zone 2		Zone 3		Zone 4		Zone 5		SPI	Mean SPI
		*N*	*D*	*N*	*D*	*N*	*D*	*N*	*D*	*N*	*D*		
Sloth	Ursula	327	309	366	72	176	−120	122	−97	59	−163	0.369	0.365
	Colombo	286	268	398	104	213	−83	133	−86	20	−201	0.360	
Brown	Wendy	97	37	64	−20	50	−107	347	150	156	−60	0.286	0.310
	Wellington	132	72	62	−22	45	−112	365	168	110	−106	0.367	
	Winslow	87	27	29	−55	30	−127	349	152	219	−3	0.278	
